# (4*S*,5*R*,6*R*)-Methyl 4-hydr­oxy-4,5-iso­propyl­idenedioxy-4,5,6,7-tetra­hydro-1,2,3-triazolo[1,5-*a*]pyridine-3-carboxyl­ate

**DOI:** 10.1107/S1600536809006357

**Published:** 2009-02-25

**Authors:** Sarah F. Jenkinson, Jennifer R. Fenton, K. Victoria Booth, George W. J. Fleet, David J. Watkin

**Affiliations:** a Department of Organic Chemistry, Chemistry Research Laboratory, Department of Chemistry, University of Oxford, Oxford OX1 3TA, England; b Department of Chemical Crystallography, Chemistry Research Laboratory, Department of Chemistry, University of Oxford, Oxford OX1 3TA, England

## Abstract

X-ray crystallography confirmed the structure of the title triazole, C_11_H_15_N_3_O_5_, formed from a single-step reaction of a sugar azide with a brominated ylid. The absolute configuration was determined by the use of d-ribose as the starting material. The six-membered ring is in a half-chair conformation. The crystal structure exists as chains of O—H⋯O hydrogen-bonded moleclues running parallel to the *b* axis.

## Related literature

For imino sugars, see: Asano *et al.* (2000 ▶); Watson *et al.* (2001 ▶). For sugar tetra­zoles, see: Brandstetter *et al.* (1995 ▶); Davis *et al.* (1995 ▶); Ermert *et al.* (1991 ▶). For sugar triazoles, see: Caravano *et al.* (2007 ▶); Krivopalov & Shkurko (2005 ▶); Krulle *et al.* (1997 ▶); Marco-Contelles & Rodriguez-Fernandez (2001 ▶, 2002 ▶); Oikonomakos (2002 ▶); Tatsuta *et al.* (1996 ▶). For related literature, see: Görbitz (1999 ▶); Larson (1970 ▶).
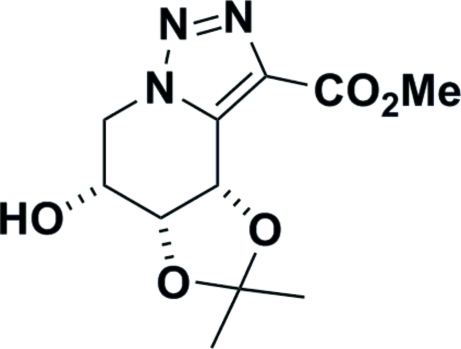


## Experimental

### 

#### Crystal data

C_11_H_15_N_3_O_5_
*M* *_r_* = 269.26
Monoclinic, 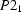

*a* = 8.0587 (3) Å
*b* = 7.3797 (3) Å
*c* = 10.9785 (5) Å
β = 96.2740 (18)°
*V* = 648.99 (5) Å^3^
*Z* = 2
Mo *K*α radiation
μ = 0.11 mm^−1^
*T* = 150 K
0.60 × 0.15 × 0.03 mm

#### Data collection

Nonius KappaCCD diffractometer
Absorption correction: multi-scan (*DENZO*/*SCALEPACK*; Otwinowski & Minor, 1997 ▶) *T*_min_ = 0.82, *T*_max_ = 1.00 (expected range = 0.817–0.997)
9525 measured reflections
1595 independent reflections
1219 reflections with *I* > 2σ(*I*)
*R*_int_ = 0.059

#### Refinement

*R*[*F* ^2^ > 2σ(*F* ^2^)] = 0.037
*wR*(*F* ^2^) = 0.082
*S* = 0.96
1595 reflections
173 parameters
1 restraint
H-atom parameters constrained
Δρ_max_ = 0.30 e Å^−3^
Δρ_min_ = −0.31 e Å^−3^

### 

Data collection: *COLLECT* (Nonius, 2001 ▶).; cell refinement: *DENZO*/*SCALEPACK* (Otwinowski & Minor, 1997 ▶); data reduction: *DENZO*/*SCALEPACK*; program(s) used to solve structure: *SIR92* (Altomare *et al.*, 1994 ▶); program(s) used to refine structure: *CRYSTALS* (Betteridge *et al.*, 2003 ▶); molecular graphics: *CAMERON* (Watkin *et al.*, 1996 ▶); software used to prepare material for publication: *CRYSTALS*.

## Supplementary Material

Crystal structure: contains datablocks global, I. DOI: 10.1107/S1600536809006357/lh2778sup1.cif

Structure factors: contains datablocks I. DOI: 10.1107/S1600536809006357/lh2778Isup2.hkl

Additional supplementary materials:  crystallographic information; 3D view; checkCIF report

## Figures and Tables

**Table 1:** Hydrogen-bond geometry (Å, °)

*D*—H⋯*A*	*D*—H	H⋯*A*	*D*⋯*A*	*D*—H⋯*A*
O19—H191⋯O4^i^	0.84	1.96	2.782 (4)	163

Symmetry code: (i) 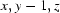.

**Fig. 1.**
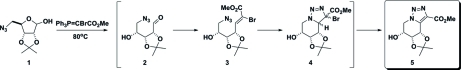
Synthetic Scheme.

**Fig. 2.**
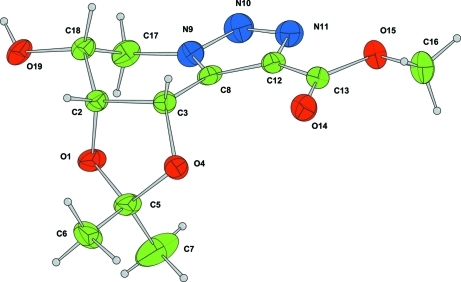
The molecluar structure showing the crystallographic labelling scheme and displacement ellipsoids drawn at the 50% probability level. H atoms are shown as spheres of arbitary radius.

**Fig. 3.**
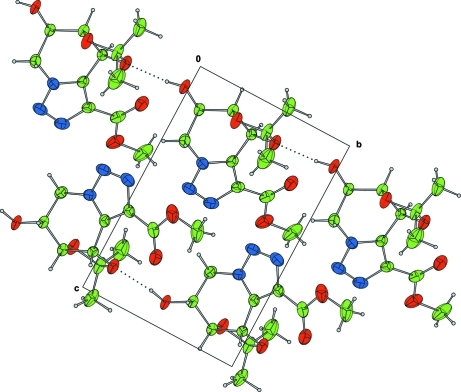
Part of the crystal structure of the title compound projected along the a-axis. Hydrogen bonds are indicated by dotted lines.

## supplementary crystallographic information

### Comment

Sugars with the ring oxygen replaced by nitrogen comprise a large family of both natural products and synthetic analogues which inhibit sugar metabolizing enzymes (Asano *et al.*, 2000; Watson *et al.*, 2001), including compounds which incorporate a tetrazole (Ermert *et al.*, 1991; Davis *et al.*, 1995; Brandstetter *et al.*, 1995) or triazole (Tatsuta *et al.*, 1996; Marco-Contelles & Rodriguez-Fernandez, 2002; Caravano *et al.*, 2007; Krivpalov & Shkurko, 2007) fused to the pyranose ring. Some sugar triazoles have potential as glycogen phosphorylase inhibitors (Oikonomakos, 2002). Usually the synthesis of pyranose triazoles requires many steps (Marco-Contelles & Rodriguez-Fernandez, 2001; Krulle *et al.*, 1997).

A single step synthesis (see Fig. 1) has been developed in which an azidolactol 1 was reacted with Ph_3_P=CBrCOOMe; the open chain form 2 underwent a Wittig reaction to give 3 which was followed by an intramolecular 1,3-dipolar addition of the azide to the alkene to afford 4. Subsequent elimination of HBr gave the target compound 5. The structure of the product 5, including the relative configuration of the three chiral centers was confirmed by X-ray crystallographic analysis. The absolute configuration was determined by the use of D-ribose as the starting material for the preparation of azidolactol 1.

The crystal structure of 5 exisits as chains of O—H···O hydrogen bonded moleclues lying parallel to the *b*-axis. Only classical hydrogen bonding has been considered. The 6-membered ring exists in a half-chair conformation.

### Experimental

The title compound was recrystallized by vapour diffusion from a mixture of ether and cyclohexane: m.p. 413–415 K; [α]_D_^21^ -140.7 (*c*, 1.01 in CHCl_3_).

### Refinement

In the absence of significant anomalous scattering, Friedel pairs were merged and the absolute configuration was assigned from the starting material.

The relatively large ratio of minimum to maximum corrections applied in the multiscan process (1:1.21) reflect changes in the illuminated volume of the crystal. Changes in illuminated volume were kept to a minimum, and were taken into account (Görbitz, 1999) by the multi-scan inter-frame scaling (*DENZO*/*SCALEPACK*, Otwinowski & Minor, 1997).

The H atoms were all located in a difference map, but those attached to carbon atoms were repositioned geometrically. The H atoms were initially refined with soft restraints on the bond lengths and angles to regularize their geometry (C—H in the range 0.93–0.98, O—H = 0.82 Å) and *U*_iso_(H) (in the range 1.2–1.5 times *U*_eq_ of the parent atom), after which the positions were refined with riding constraints.

### Crystal data

**Table:**

C_11_H_15_N_3_O_5_	*F*(000) = 284
*M**_r_* = 269.26	*D*_x_ = 1.378 Mg m^−^^3^
Monoclinic, *P*2_1_	Mo *K*α radiation, λ = 0.71073 Å
Hall symbol: P 2yb	Cell parameters from 1565 reflections
*a* = 8.0587 (3) Å	θ = 5–27°
*b* = 7.3797 (3) Å	µ = 0.11 mm^−^^1^
*c* = 10.9785 (5) Å	*T* = 150 K
β = 96.2740 (18)°	Plate, colourless
*V* = 648.99 (5) Å^3^	0.60 × 0.15 × 0.03 mm
*Z* = 2	

### Data collection

**Table:**

Nonius KappaCCD diffractometer	1219 reflections with *I* > 2σ(*I*)
graphite	*R*_int_ = 0.059
ω scans	θ_max_ = 27.5°, θ_min_ = 5.2°
Absorption correction: multi-scan (*DENZO*/*SCALEPACK*; Otwinowski & Minor, 1997)	*h* = −10→10
*T*_min_ = 0.82, *T*_max_ = 1.00	*k* = −9→9
9525 measured reflections	*l* = −14→14
1595 independent reflections	

### Refinement

**Table:**

Refinement on *F*^2^	Hydrogen site location: inferred from neighbouring sites
Least-squares matrix: full	H-atom parameters constrained
*R*[*F*^2^ > 2σ(*F*^2^)] = 0.037	Method = Modified Sheldrick *w* = 1/[σ^2^(*F*^2^) + (0.04*P*)^2^ + 0.06*P*], where *P* = [max(*F*_o_^2^,0) + 2*F*_c_^2^]/3
*wR*(*F*^2^) = 0.082	(Δ/σ)_max_ = 0.0001
*S* = 0.97	Δρ_max_ = 0.30 e Å^−^^3^
1595 reflections	Δρ_min_ = −0.31 e Å^−^^3^
173 parameters	Extinction correction: Larson (1970), Equation 22
1 restraint	Extinction coefficient: 120 (30)
Primary atom site location: structure-invariant direct methods	

### Fractional atomic coordinates and isotropic or equivalent isotropic displacement parameters (Å^2^)

**Table:**

	*x*	*y*	*z*	*U*_iso_*/*U*_eq_	
O1	0.2044 (2)	0.3385 (2)	0.12370 (17)	0.0348	
C2	0.3680 (3)	0.2921 (3)	0.0939 (2)	0.0259	
C3	0.4700 (3)	0.4559 (3)	0.14365 (19)	0.0263	
O4	0.3535 (2)	0.6018 (2)	0.12971 (16)	0.0369	
C5	0.1853 (3)	0.5296 (3)	0.1071 (3)	0.0371	
C6	0.1173 (3)	0.5757 (4)	−0.0230 (3)	0.0481	
C7	0.0820 (4)	0.5983 (5)	0.2022 (3)	0.0711	
C8	0.5404 (3)	0.4306 (3)	0.27502 (19)	0.0274	
N9	0.5115 (2)	0.2809 (3)	0.33823 (15)	0.0314	
N10	0.5960 (3)	0.2829 (4)	0.45255 (16)	0.0404	
N11	0.6780 (2)	0.4367 (3)	0.46271 (17)	0.0393	
C12	0.6470 (3)	0.5312 (3)	0.35555 (19)	0.0301	
C13	0.7152 (3)	0.7105 (4)	0.3339 (2)	0.0347	
O14	0.6759 (2)	0.7985 (3)	0.24224 (16)	0.0410	
O15	0.8253 (2)	0.7650 (3)	0.42672 (17)	0.0493	
C16	0.8993 (4)	0.9424 (5)	0.4125 (3)	0.0619	
C17	0.4111 (3)	0.1253 (4)	0.2922 (2)	0.0340	
C18	0.4225 (3)	0.1151 (3)	0.1552 (2)	0.0282	
O19	0.3191 (2)	−0.0250 (2)	0.10143 (15)	0.0345	
H21	0.3716	0.2823	0.0030	0.0326*	
H31	0.5628	0.4800	0.0923	0.0335*	
H62	0.1213	0.7081	−0.0306	0.0684*	
H61	0.0013	0.5339	−0.0390	0.0679*	
H63	0.1873	0.5166	−0.0791	0.0683*	
H72	0.0760	0.7296	0.1946	0.1122*	
H71	−0.0294	0.5466	0.1898	0.1121*	
H73	0.1367	0.5658	0.2826	0.1119*	
H163	0.9999	0.9506	0.4700	0.0913*	
H162	0.9284	0.9553	0.3294	0.0911*	
H161	0.8193	1.0353	0.4304	0.0913*	
H172	0.2930	0.1423	0.3074	0.0432*	
H171	0.4592	0.0151	0.3327	0.0435*	
H181	0.5417	0.0915	0.1425	0.0336*	
H191	0.3489	−0.1327	0.1166	0.0521*	

### Atomic displacement parameters (Å^2^)

**Table:**

	*U*^11^	*U*^22^	*U*^33^	*U*^12^	*U*^13^	*U*^23^
O1	0.0270 (8)	0.0198 (9)	0.0582 (11)	0.0004 (7)	0.0067 (7)	0.0020 (7)
C2	0.0241 (11)	0.0212 (11)	0.0325 (11)	0.0008 (10)	0.0032 (8)	−0.0010 (11)
C3	0.0298 (11)	0.0224 (12)	0.0259 (11)	0.0021 (10)	−0.0007 (9)	0.0011 (10)
O4	0.0340 (9)	0.0205 (9)	0.0524 (11)	0.0020 (8)	−0.0125 (7)	−0.0004 (8)
C5	0.0267 (12)	0.0178 (12)	0.0658 (17)	0.0013 (10)	0.0007 (11)	−0.0001 (12)
C6	0.0357 (13)	0.0268 (14)	0.0762 (19)	−0.0026 (12)	−0.0191 (13)	0.0064 (14)
C7	0.075 (2)	0.041 (2)	0.105 (3)	0.0097 (18)	0.0425 (19)	−0.0053 (19)
C8	0.0270 (11)	0.0247 (12)	0.0305 (11)	0.0084 (11)	0.0040 (9)	−0.0028 (11)
N9	0.0372 (10)	0.0314 (11)	0.0256 (9)	0.0035 (10)	0.0040 (8)	0.0024 (9)
N10	0.0491 (12)	0.0475 (14)	0.0241 (9)	0.0073 (12)	0.0023 (8)	0.0009 (11)
N11	0.0427 (11)	0.0465 (14)	0.0279 (10)	0.0110 (12)	−0.0003 (8)	−0.0067 (11)
C12	0.0294 (11)	0.0315 (14)	0.0283 (12)	0.0091 (10)	−0.0011 (9)	−0.0061 (11)
C13	0.0262 (11)	0.0346 (14)	0.0413 (14)	0.0068 (11)	−0.0055 (10)	−0.0129 (12)
O14	0.0382 (9)	0.0298 (10)	0.0524 (11)	0.0004 (9)	−0.0062 (8)	−0.0029 (10)
O15	0.0406 (10)	0.0451 (13)	0.0576 (11)	0.0030 (9)	−0.0157 (8)	−0.0195 (10)
C16	0.0454 (15)	0.0440 (19)	0.091 (2)	−0.0055 (16)	−0.0167 (14)	−0.0262 (18)
C17	0.0404 (13)	0.0262 (13)	0.0366 (13)	0.0005 (11)	0.0099 (10)	0.0045 (11)
C18	0.0313 (11)	0.0196 (12)	0.0335 (12)	0.0020 (10)	0.0030 (9)	0.0005 (10)
O19	0.0413 (9)	0.0137 (8)	0.0481 (10)	0.0003 (7)	0.0036 (7)	−0.0011 (7)

### Geometric parameters (Å, °)

**Table:**

O1—C2	1.434 (3)	C8—C12	1.380 (3)
O1—C5	1.429 (3)	N9—N10	1.361 (3)
C2—C3	1.529 (3)	N9—C17	1.463 (3)
C2—C18	1.512 (3)	N10—N11	1.312 (3)
C2—H21	1.004	N11—C12	1.367 (3)
C3—O4	1.426 (3)	C12—C13	1.462 (4)
C3—C8	1.503 (3)	C13—O14	1.211 (3)
C3—H31	1.000	C13—O15	1.338 (3)
O4—C5	1.452 (3)	O15—C16	1.454 (4)
C5—C6	1.512 (4)	C16—H163	0.973
C5—C7	1.494 (4)	C16—H162	0.971
C6—H62	0.981	C16—H161	0.976
C6—H61	0.982	C17—C18	1.519 (3)
C6—H63	0.982	C17—H172	0.992
C7—H72	0.973	C17—H171	0.986
C7—H71	0.971	C18—O19	1.416 (3)
C7—H73	0.973	C18—H181	1.001
C8—N9	1.339 (3)	O19—H191	0.842
			
C2—O1—C5	107.18 (19)	C3—C8—C12	133.8 (2)
O1—C2—C3	101.66 (18)	N9—C8—C12	104.07 (19)
O1—C2—C18	109.52 (18)	C8—N9—N10	111.8 (2)
C3—C2—C18	113.92 (17)	C8—N9—C17	126.16 (18)
O1—C2—H21	111.8	N10—N9—C17	122.0 (2)
C3—C2—H21	109.8	N9—N10—N11	106.53 (19)
C18—C2—H21	110.0	N10—N11—C12	108.96 (19)
C2—C3—O4	103.68 (16)	C8—C12—N11	108.7 (2)
C2—C3—C8	112.1 (2)	C8—C12—C13	127.0 (2)
O4—C3—C8	111.83 (18)	N11—C12—C13	124.4 (2)
C2—C3—H31	110.2	C12—C13—O14	123.5 (2)
O4—C3—H31	109.2	C12—C13—O15	112.2 (2)
C8—C3—H31	109.6	O14—C13—O15	124.3 (3)
C3—O4—C5	109.44 (17)	C13—O15—C16	115.8 (2)
O4—C5—O1	104.75 (19)	O15—C16—H163	108.0
O4—C5—C6	108.3 (2)	O15—C16—H162	109.4
O1—C5—C6	111.5 (2)	H163—C16—H162	109.5
O4—C5—C7	109.7 (2)	O15—C16—H161	108.8
O1—C5—C7	107.8 (2)	H163—C16—H161	110.4
C6—C5—C7	114.3 (2)	H162—C16—H161	110.6
C5—C6—H62	107.0	N9—C17—C18	106.86 (19)
C5—C6—H61	109.7	N9—C17—H172	110.3
H62—C6—H61	109.7	C18—C17—H172	109.6
C5—C6—H63	108.6	N9—C17—H171	108.5
H62—C6—H63	111.3	C18—C17—H171	110.0
H61—C6—H63	110.4	H172—C17—H171	111.4
C5—C7—H72	107.7	C17—C18—C2	110.60 (19)
C5—C7—H71	110.2	C17—C18—O19	110.65 (19)
H72—C7—H71	110.0	C2—C18—O19	108.43 (17)
C5—C7—H73	108.6	C17—C18—H181	108.0
H72—C7—H73	109.7	C2—C18—H181	108.9
H71—C7—H73	110.5	O19—C18—H181	110.2
C3—C8—N9	122.1 (2)	C18—O19—H191	117.7

### Hydrogen-bond geometry (Å, °)

**Table:**

*D*—H···*A*	*D*—H	H···*A*	*D*···*A*	*D*—H···*A*
C3—H31···O19^i^	1.00	2.42	3.339 (4)	152
C16—H161···N10^ii^	0.98	2.59	3.567 (4)	174
O19—H191···O4^iii^	0.84	1.96	2.782 (4)	163

Symmetry codes: (i) −*x*+1, *y*+1/2, −*z*; (ii) *x*, *y*+1, *z*; (iii) *x*, *y*−1, *z*.
