# (4*S*,5*R*,6*R*)-Methyl 4-hydr­oxy-4,5-iso­propyl­idenedioxy-4,5,6,7-tetra­hydro-1,2,3-triazolo[1,5-*a*]pyridine-3-carboxyl­ate. Erratum

**DOI:** 10.1107/S1600536809030529

**Published:** 2009-08-29

**Authors:** Sarah F. Jenkinson, Jennifer R. Fenton, K. Victoria Booth, George W. J. Fleet, David J. Watkin

**Affiliations:** a Department of Organic Chemistry, Chemistry Research Laboratory, Department of Chemistry, University of Oxford, Oxford OX1 3TA, England; b Department of Chemical Crystallography, Chemistry Research Laboratory, Department of Chemistry, University of Oxford, Oxford OX1 3TA, England

## Abstract

The chemical name of the title compound in the paper by Jenkinson, Fenton, Booth, Fleet & Watkin [*Acta Cryst.* (2009), E**65**, o610–o611] is corrected.

## Abstract 2

Corrigendum to *Acta Cryst.* (2009), E**65**, o610–o611.

In the paper by Jenkinson, Fenton, Booth, Fleet & Watkin [*Acta Cryst.* (2009), E**65**, o610–o611], the chemical name given in the *Title* should be ‘(4*S*,5*R*,6*R*)-Methyl 6-hydr­oxy-4,5-iso­propyl­idenedioxy-4,5,6,7-tetra­hydro-1,2,3-triazolo[1,5-*a*]pyri­dine-3-carboxyl­ate’.

